# Investigating the performance of a novel pH and cathepsin B sensitive, stimulus-responsive nanoparticle for optimised sonodynamic therapy in prostate cancer

**DOI:** 10.1016/j.jconrel.2020.11.040

**Published:** 2021-01-10

**Authors:** Marym Mohammad Hadi, Heather Nesbitt, Hamzah Masood, Fabiola Sciscione, Shiv Patel, Bala S. Ramesh, Mark Emberton, John F. Callan, Alexander MacRobert, Anthony P. McHale, Nikolitsa Nomikou

**Affiliations:** a Division of Surgery & Interventional Science, Faculty of Medical Sciences, University College London, UK; b Biomedical Sciences Research Institute, Ulster University, Coleraine, UK

**Keywords:** Sonodynamic therapy, Nanoparticles, Sensitizer, Prostate cancer, Cathepsin B, Tumour microenvironment

## Abstract

Nano-formulations that are responsive to tumour-related and externally-applied stimuli can offer improved, site-specific antitumor effects, and can improve the efficacy of conventional therapeutic agents. Here, we describe the performance of a novel stimulus-responsive nanoparticulate platform for the targeted treatment of prostate cancer using sonodynamic therapy (SDT). The nanoparticles were prepared by self-assembly of poly(L-glutamic acid-L-tyrosine) *co*-polymer with hematoporphyrin. The nanoparticulate formulation was characterized with respect to particle size, morphology, surface charge and singlet oxygen production during ultrasound exposure. The response of the formulation to the presence of cathepsin B, a proteolytic enzyme that is overexpressed and secreted in the tumour microenvironment of many solid tumours, was assessed. Our results showed that digestion with cathepsin B led to nanoparticle size reduction. In the absence of ultrasound, the formulation exhibited greater toxicity at acidic pH than at physiological pH, using the human prostate cells lines LNCaP and PC3 as targets. Nanoparticle cellular uptake was enhanced at acidic pH – a condition that was also associated with greater cathepsin B production. Nanoparticles exhibited enhanced ultrasound-induced cytotoxicity against both prostate cancer cell lines. Subsequent proof-of-concept *in vivo* studies demonstrated that, when ectopic human xenograft LNCaP tumours in SCID mice were treated with SDT using the systemically-administered nanoparticulate formulation at a single dose, tumour volumes decreased by up to 64% within 24 h. No adverse effects were observed in the nanoparticle-treated mice and their body weight remained stable. The potential of this novel formulation to deliver safe and effective treatment of prostate cancer is discussed.

## Highlights

• Digestion by cathepsin B leads to nanoparticle size reduction.
• The acidic pH facilitates improved cellular uptake of the nanoparticles.
• Ultrasound–induced cytotoxic effects were elicited only for the nanoparticle-treated prostate cancer cells.
• Sonodynamic treatment resulted in an average of 36% reduction in prostate tumour volume, within 24 h.

## Graphical abstract

**Figure:**
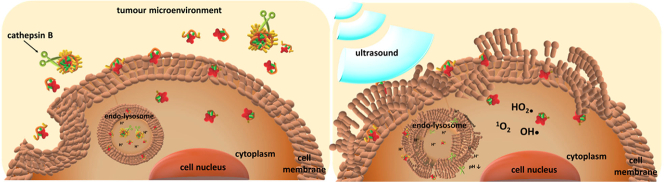

## 1 Introduction

**Fig. 1:**
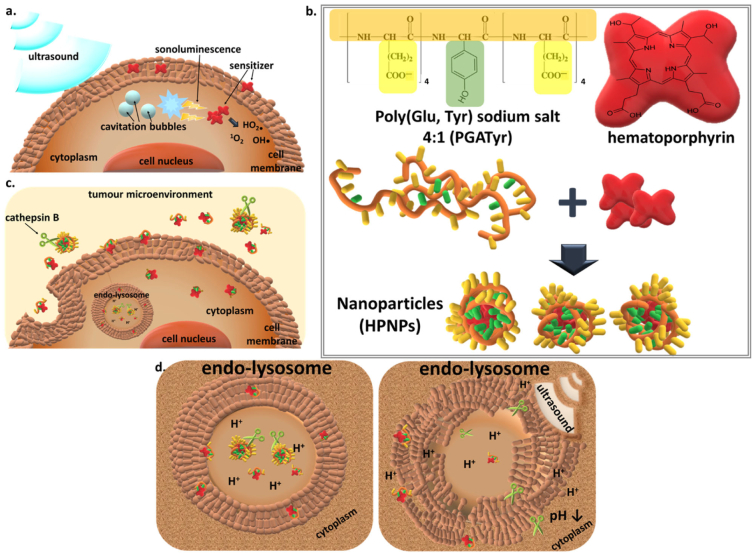
Illustration of (a) the suggested mechanism of the cytotoxic effect induced by SDT: cavitation bubbles formed upon ultrasound irradiation implode and emit sonoluminescence that excites the sensitizer leading to the production of cytotoxic ROS, (b) self assembly of the *co*-polymer with hematoporphyrin, *via* hydrophobic and π-π interactions, for the formation of HPNP nanoparticles, (c) cathepsin B in the tumour extracellular environment digests HPNPs, leading to the formation of smaller particles, improved diffusion within the tumour mass and improved cellular uptake, and (d) HPNP digestion by cathepsin B in the *endo*-lysosome can promote the incorporation of free hematoporphyrin or amphiphilic hematoporphyrin complexes in the lysosomal membrane, the sensitization of the latter and the subsequent lysosome collapse upon ultrasound irradiation, due to sonochemical effects.

Sonodynamic therapy (SDT) employs low-intensity ultrasound in combination with sensitizing agents, such as porphyrins, for the generation of cytotoxic reactive oxygen species (ROS) and the subsequent site-specific ablation of tumours [1]. Ultrasound can be applied locally, either extracorporeally or by endoscopic / transrectal intervention, and the acoustic waves, at frequencies between 0.5 and 2 MHz, are transmitted efficiently through tissue to irradiate the target tumour, as long as there is a suitable “acoustic window” between the ultrasound transducer and the target [2]. It has been reported that, when tissue is exposed to ultrasound, at this relevant frequency range, gas micro- and nano-bubbles are nucleated, a phenomenon known as cavitation. Under ultrasound exposure, these bubbles oscillate and eventually collapse (inertial cavitation), producing light, a phenomenon known as sonoluminscence [3,4]. Since many sonosensitisers are also photosensitisers, it has been suggested that the production of ROS during SDT is due to photoexcitation of the sensitizer by sonoluminescence (Fig. 1a). The latter excites the sensitizing agent and this phenomenon results in the production of either cytotoxic singlet oxygen (^1^O_2_) by intersystem crossing and direct energy transfer (Type II reactions) or to the generation of other cytotoxic free radicals by electron transfer to other molecular species such as biological molecules (Type I reactions) [5].

SDT does not require the systemic administration of potentially toxic agents and the cytotoxic effect tends to be limited to the target tissue as it is dependent on exposure to ultrasound energy above a given threshold. Further highlighting the benefits of SDT, no cancer cell population has shown resistance to therapy-triggered ROS production or their cytotoxic effects [6]. This is particularly important, given the as yet unresolved issues of radiation and chemotherapy associated resistance.

A number of relatively recent reports have demonstrated that the efficacy and translational potential of SDT, for a broad range of cancers, can be augmented with nanotechnology. Various nanoparticulate designs have been employed to serve as carriers of sensitizers, such as hematoporphyrin and its derivatives. The relevant preclinical studies have shown that these formulations can improve the antitumour effect of sensitizers in response to ultrasound in a multifunctional manner. Apart from improving the bioavailability and tumour accumulation of relatively hydrophobic sensitizers, nanoformulation has the potential to potentiate sonoactivation of sensitizers, improve cellular uptake within tumours and even co-carry agents with synergistic antitumour effects, such as immunoadjuvants or immune checkpoint inhibitors. Polymeric nanoparticles or micelles have been designed as carriers for the systemic administration of hydrophobic sensitizers, such as hematoporphyrin [6]. Nanoparticulate formulations with porous structures have been developed to seed bubble formation upon ultrasound irradiation and augment sonoluminescence-induced excitation of the sensitizing agent for the production of ROS [7,8].

Multi-stimulus-responsive anticancer nano-formulations can promote drug release and activation within the target tumour, facilitate cellular uptake, as well as improve the therapeutic efficacy of drugs. In solid tumours disease-specific stimulation can be provoked by the well-known distinctive characteristics of the tumour microenvironment, such as hypoxia, the acidic pH, the enhanced permeability and retention (EPR) effect, the abundance of proteolytic enzymes, and the overexpression of particular cell membrane antigens or proteolytic factors. These endogenous, tumour-specific characteristics provide valuable tools for the design of formulations with enhanced activity in the tumour microenvironment. Polyglutamate (PGA) is among the biodegradable polymers that have been successfully used in drug delivery systems for cancer, mainly as PGA-drug conjugates [9]. Interestingly, PGA can be digested by cathepsin-B, which is a lysosomal protease that is hyper-secreted into the tumour microenvironment of many solid tumours and we and others have demonstrated that this can be exploited in targeted therapeutic approaches [10,11].

Below, we describe the development and initial testing of a nanoparticulate hematoporphyrin-carrying formulation, based on a *co*-polymer of glutamate and tyrosine. The nanoparticles are designed to improve the delivery of hematoporphyrin to prostate tumours, its diffusion throughout the dense tumour mass and its efficacy in augmenting ultrasound-induced targeted toxicity. We report on the response of the formulation to particular characteristics of the prostate tumour microenvironment and its efficacy in mediating SDT-based cytotoxic effects under these conditions *in vitro*. In addition, proof-of-concept studies were performed in order to demonstrate nanoparticle-mediated SDT efficacy using an ectopic human prostate cancer xenograft model in mice.

## 2 Materials and methods

### 2.1 *Co*-polymer digestion with Cathepsin-B

The poly(L-glutamic acid-L-tyrosine) 4:1, sodium salt (PGATyr) (MW: 20–50 kDa; Sigma-Aldrich, UK) (2 mg/mL) was incubated with cathepsin-B (from human placenta; Sigma-Aldrich, UK) (0.6 units/mL) in pH 6.4 and pH 7.4 solutions, for 3 days, in an orbital incubator, at 37 °C. 15 μ*L* aliquots were obtained from the incubating mixture every 24 h and were stored at −20 °C until use. Samples were treated with tris-glycine sodium dodecyl sulphate (SDS) sample buffer (Novex by Life Technologies, UK) as recommended by the manufacturer prior to gradient SDS/ polyacrylamide gradient gel electrophoresis (PAGE). After electrophoresis, gels were stained with 0.5% Brilliant Blue G-Colloidal concentrate (Sigma-Aldrich, UK) for 24 h to expose the protein ladder bands, followed by staining with 0.5% Alcian blue (BDH chemicals, UK) for visualising PGATyr. The gel bands were analyzed using ImageJ® for determining the progress of digestion within the course of 3 days.

### 2.2 Preparation of hematoporphyrin-containing PGATyr-based nanoparticles

For the preparation of the hematoporphyrin-containing PGATyr-based nanoparticles (HPNPs), 10 mg of PGATyr and 5 mg of hematoporphyrin (HP) (Sigma Aldrich, UK) were dissolved in 10 mL of dimethyl sulfoxide (DMSO) (Sigma-Aldrich, UK). The resulting mixture was added drop-wise to a 5 mL polyvinyl alcohol solution (PVA, MW: 124 kDa, Sigma-Aldrich, UK) (0.5 mg/mL) and the mixture was left under constant stirring for 1 h. The mixture was then dialysed against water using dialysis tubing (MWCO: 8 kDa, Theromofisher Scientific), for 24 h. Subsequently, the suspension was centrifuged for 90 min at 38,000*g*, at 12 °C using a Beckman OptimaTM L-80 ultracentrifuge. The precipitated pellet was suspended in 2 mL deionised water and was probe-sonicated for 3 min, followed by further dialysis against deionised water for 12 h. The dialysed solution was then sterile-filtered using a micro syringe filter (0.22 μm, Millex GP). The suspension was snap-frozen in a sterile round-bottom flask and freeze dried for 24 h. The dried sample was resuspended in 3 mL phosphate buffered saline (PBS) and was stored at 4 °C, protected from light.

### 2.3 HPNP characterization and the effect of digestion with cathepsin B

The HP concentration in the HPNP suspension was determined using UV–Vis spectroscopy, by reference to a standard curve for HP, in a 1:9 mixture of PBS and DMSO, at 534 nm. The loading efficiency of HP (or the yield) for the nanoparticle preparation was calculated as the weight ratio of the total HP in the final suspension to the quantity of initially added HP for the preparation of the formulation. A Zeiss EVO MA10 Field Emission Scanning Electron Microscope was used to determine the nanoparticle size and morphology. The mean hydrodynamic diameter of the undigested and digested nanoparticles was determined by dynamic light scattering (DLS) using a DelsaMax PRO Particle Analyzer (Beckman Coulter, UK). For the preparation of the digested samples, the nanoparticles were incubated with cathepsin B (0.2 units/mL), at pH 6.4 and pH 7.4 (in buffered suspensions), at 37 °C, for 24 h. The size of nanoparticles in the presence of 10% fetal bovine serum (FBS) (Life Technologies, UK) was also measured at varying pH values. The surface charge of the digested and undigested nanoparticles was measured with electrophoretic light scattering using a Zetasizer Ultra (Malvern Instruments, UK).

For the quantitative analysis of ROS, except for hydrogen peroxide [12], free HP or HPNPs were suspended in PBS containing 10% *v*/v FBS. The suspensions were then mixed, at a 2:1 ratio, with a 1,3-diphenylisobenzofuran (DPBF) solution 0.4 mM in ethanol, to give a final HP concentration of 20 μM (12 μg/mL), in a total volume of 90 mL. The systems were exposed to ultrasound by immersing the ultrasound transducer of a Sonidel SP100 sonoporator (Sonidel Ltd., Ireland) into the 90 mL suspension. Ultrasound was applied at a frequency of 1 MHz, a power density (intensity) of 4 W/cm^2^ and 25% duty cycle using a pulse repetition frequency of 100 Hz. The system was protected from light. During ultrasound exposure, 3.5 mL aliquots were collected at different times (0, 1, 3 and 5 min) and were centrifuged for 1 min. The absorbance spectra of the supernatants were then obtained at wavelengths between 380 and 460 nm using a UV/Vis spectrophotometer (JASCO Corporation, Japan). Since HP absorbs a considerable amount of light in the wavelengths of interest, the corresponding systems were also run in the absence of DPBF. The absorbance values of the DPBF-free samples were subtracted from those of the corresponding test samples (containing DPBF), resulting in a single absorption peak at approx. 436 nm for time point 0 min.

### 2.4 Cell culture

The human-derived prostate cancer cell lines LNCaP and PC3 were used in this study. LNCaP cells were obtained from the American Type Culture Collection (USA) and PC3 cells were obtained from Public Health England. These cell lines were selected on the basis of their aggressive nature and their differential PSMA expression (LNCaP: PSMA +ve, PC3: PSMA –ve). Both cell lines were maintained in RPMI 1640 Medium (1×) (Life Technologies) supplemented with l-glutamine (GibcoBRL, UK), 10% *v*/v (FBS and 1% penicillin & streptomycin (Sigma-Aldrich, UK), at 37 °C, in a 5% CO_2_ humidified atmosphere. When required, single cell suspensions were prepared by treating cell monolayers with a 0.05% *w*/*v* solution of trypsin containing 0.02% w/v EDTA in PBS. Incubation at pH 6.4 was performed in growth medium by the 0.15% *v*/v addition of lactic acid 85% w/v solution. The cell viability was determined indirectly by measuring the relative metabolic activity using an MTT assay [13].

### 2.5 “Silent” toxicity of the nanoparticles and free HP

For determining the toxicity of the nanoparticles and the free hematoporphyrin, in the absence of ultrasound irradiation (*i.e.* the “silent” toxicity), LNCaP and PC3 cells were seeded in wells of 96 well-plates at a concentration of 15 × 10^3^ cells per well and incubated, under normoxic conditions, for 24 h. The growth medium was then replaced with complete (including FBS) growth medium containing either HPNPs or free HP, at either pH 6.4 (adjusted as described in Section 2.3) or pH 7.4 (physiological pH of growth medium), and at concentrations ranging from 0.01 to 20 μg/mL based on HP. The control systems contained growth medium at the corresponding pH, in the absence of a sensitizing agent. Cells were incubated for 24 h after which media were removed, cell monolayers were washed with PBS and fresh growth medium was added. Plates were then incubated for a further 24 h and cell viability was determined using the MTT assay.

### 2.6 Intracellular and extracellular cathepsin B activity

LNCaP cells were seeded in wells of 24 well-plates at a concentration of 6 × 10^4^ cells per well and incubated for 24 h under normoxic conditions. The growth medium was then removed, and the cells were incubated in a hypoxic (2 mmHg O_2_) environment, at pH 6.4 or pH 7.4, for 24 h. Intracellular and extracellular cathepsin B activity was determined using a fluorometric cathepsin B activity assay kit (Abcam, UK), from lysed cells and the corresponding growth medium, respectively. The incubation medium was collected for extracellular cathepsin B analysis. For the intracellular cathepsin B assessment, cells were lysed using a cell lysis buffer (Abcam, UK) according to the manufacturer's instructions. Subsequently, 50 μL aliquots of each cell lysate or extracellular medium sample were mixed with 50 μL of cathepsin B reaction buffer and 2 μL of cathepsin B substrate. Samples were incubated for 45 min and fluorescence was measured (Ex/Em = 400/505 nm). The data were normalised using the total protein concentration (mg/mL) in the cell lysate or extracellular medium samples.

### 2.7 Cellular uptake studies

To study the cellular uptake of both HP and HPNP, as well as the effect of cathepsin B on cellular uptake, LNCaP and PC3 cells were seeded in individual wells of 96-well plates at a concentration of 2 × 10^4^ cells per well. 24 h later, each HP formulation (free HP or HPNP) was added in FBS- containing growth medium (at pH 6.4 or pH 7.4) in each well. The free HP or HPNP concentration for each cell line was determined from the “silent” toxicity curves (Section 2.5) and that was a concentration with relatively low toxicity at both pH conditions. The effects of externally added cathepsin B and E64, a cathepsin B inhibitor, on cellular uptake were also examined. These molecules were added at a final concentration of 1.2 units/mL and 115 μg/mL, respectively. All systems were incubated in a hypoxic (2 mmHg O_2_) environment. The cell monolayers were then washed twice with PBS, 100 μL DMSO was added and the systems were incubated for 45 min, followed by pipette-mixing. The samples were examined using a fluorescence spectrophotometer, at 534 nm and 626 nm excitation and emission wavelengths, respectively. The fluorescence emission values were normalised against the control wells (in the absence of free HP or HPNPs) and on the basis of the cell viability in the corresponding systems determined using the MTT assay.

### 2.8 *In vitro* sonodynamic effect

LNCaP and PC3 cells were seeded in wells of 96-well plates at a concentration of 2 × 10^4^ cells per well and incubated for 24 h. Cells were then incubated in the presence or absence of HPNP or free HP, at varying concentrations, based on the cell line and the pH, determined using the “silent” toxicity profiles as indicated. All systems were incubated in hypoxic (2 mmHg O_2_) environment for 24 h. The treatment suspensions were then removed, the cell monolayers were washed with PBS and fresh growth medium was added. Cells were treated with ultrasound at a frequency of 1 MHz, at varying power density (intensity) and with varying duty cycle (DC, %) at a pulse repetition frequency of 100 Hz for 30 s using an SP100 sonoporator. Ultrasound gel was used to mediate the contact between the ultrasound transducer and the bottom of each well of the 96-well plates. Cells were then incubated for 24 h, under hypoxic conditions (2 mmHg O_2_) and the cell viability was determined using the MTT assay.

### 2.9 *In vivo* SDT treatment

All animals were treated humanely and in accordance with licenced procedures under the Animals (Scientific Procedures) Act 1986 Amendment Regulations 2012 (ASPA 2012). In these studies a human prostate xenograft model (LNCaP) in immunocompromised mice was employed. For the induction of tumours, 5 × 10^6^ LNCaP cells in 100 μL Matrigel® were injected subcutaneously into the rear dorsum of BALB/c SCID male mice (8 weeks old) using a 21-gauge needle. Tumour development was monitored over time using Vernier callipers and tumour volume was calculated using the equation: tumour volume = length * width * height/2. When the tumours had reached an average size of 85 cm^3^, animals were randomly distributed into 4 groups (*n* = 4), using the randomized allocation function of the Peira TM900 management software. Following induction of anaesthesia (intraperitoneal injection of Hypnorm/Hypnovel), a 100 μL aliquot of the HPNPs (1.5 mg/mL based on HP) was injected though the tail vein at a dose of 6 mg/kg. 24 h post-injection, the tumours were treated with ultrasound at a frequency of 1 MHz, a power density of 3.5 W/cm^2^, using a 30% duty cycle (100 Hz pulse repetition frequency), for 3.5 min. The ultrasound treatment parameters were optimised in the context of preliminary and previously published *in vivo* work [6,13]. Two additional groups of animals were treated either with the HPNPs and without ultrasound irradiation, or with ultrasound irradiation and injection of PBS. Control animals were treated with a PBS injection and tumour volume was monitored at the indicated times. The % increase in tumour volume was calculated employing the pre-treatment measurements for each group.

### 2.10 Statistical analysis

GraphPad Prism version 5 was employed for the data presentation and analysis, unless otherwise stated. Statistical analysis of significance was performed using analysis of the means and comparison of data groups. The latter was performed using one-way Anova with Tukey post-test for column statistics and two-way ANOVA with Bonferroni post-test for grouped statistics. Error bars represent ± the standard deviation (SD). A single asterisk (*) indicates *p* *<* *0.05*, double asterisk (**) indicates *p* *<* *0.01* and triple asterisk (***) indicates *p* *<* *0.001*.

## 3 Results and discussion

### 3.1 *Co*-polymer digestion with cathepsin-B

**Fig. 2:**
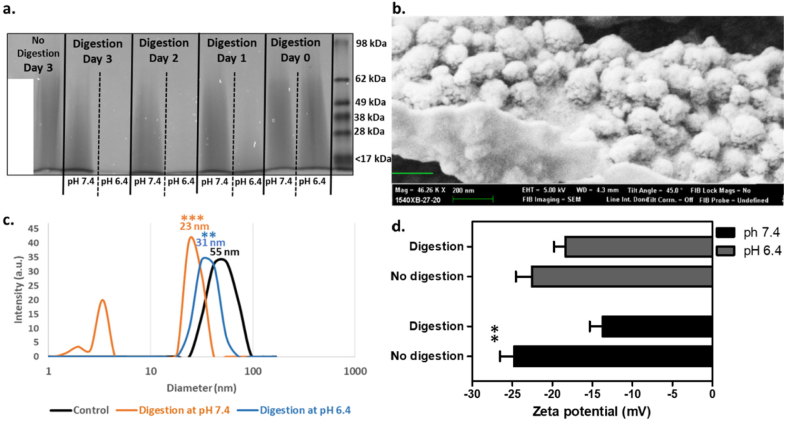
a. SDS-PAGE for PGATyr co-polymer digested by cathepsin B at 37 °C over 3 days, at pH 6.4 and pH 7.4. b. SEM image of the hematoporphyrin-carrying PGATyr-based nanoparticles (HPNPs). A size bar of 200 nm is indicated in green. c. Size distribution of undigested HPNPs (control), HPNPs digested at pH 6.4 and pH 7.4, for 24 h. The data are the average of size distribution from multiple measurements (*n* = 5) and the X-axes is at log_10_ scale. The *p* values show the significant difference between the mean diameter of the digested samples and the control (undigested). d. Zeta potential of the undigested and digested HPNPs at pH 6.4 and pH 7.4. The p value show the significant difference between digested and undigested samples at pH 7.4. Error pars represent the SEM, **p < 0.001 and ***p < 0.0001 (n = 5). (For interpretation of the references to colour in this figure legend, the reader is referred to the web version of this article.)

In a previous study, we demonstrated that PGA is efficiently cleaved by cathepsin B, an enzyme that is overexpressed and secreted by cancer cells in the tumour microenvironment [10,11]. In order to assess the effect of cathepsin B on the PGATyr *co*-polymer, the latter was incubated in the presence or the absence of the enzyme at 37 °C, at pH 6.4 and pH 7.4, over the course of 3 days. Aliquots obtained every 24 h were subjected to SDS/PAGE analysis and the image of the resolving gel is shown in Fig. 2a. The PGATyr samples incubated in the absence of cathepsin B showed a smeared profile with molecular weights extending from 70 kDa to less than 10 kDa. The smeared profile, in terms of breadth and intensity, remained unchanged, even after a 3-day incubation period, in the absence of cathepsin B. In the presence of cathepsin B, the smeared profile shifted towards lower molecular weights, when samples were digested at pH 7.4 (Supplementary Data, Fig. S1a). At pH 6.4, the intensity of the band was considerably reduced after 24 h and at that stage, the reduction of the mean molecular weight (MW) was 11-fold higher (*p* < 0.001) that that at pH 7.4 (Supplementary Data, Fig. S1). Also, the *co*-polymer appeared to be fully digested below 17 kDa after 48 h at pH 6.4. In addition to verifying that the PGATyr remained stable in the absence of cathepsin B over 3 days, these results also verified that the co-polymer, similarly to PGA, was sensitive to cathepsin B and that digestion was more efficient at acidic pH, which represents the conditions of the tumour microenvironment [14].

### 3.2 Nanoparticle characterization and the effect of digestion with cathepsin B

The HPNP structure formation was based on the self-assembly of the amphiphilic PGATyr copolymer and its complexation with HP (Fig. 1b), which was effectively supported *via* hydrophobic and π-π interactions with the tyrosine aromatic side chain of the copolymer [15]. The preparation protocol and the component ratio applied in this study resulted in a 21.3 ± 0.6% (*n* = 9) entrapment of HP (yield). Images from scanning electron microscopy (SEM) showed nanoparticles with globular morphology (Fig. 2b). The mean hydrodynamic diameter of the undigested nanoparticles, determined using DLS, was 55 nm, with nanoparticle diameter ranging from 31 nm to 96 nm (*n* = 5) (Fig. 2c) and this was confirmed by the SEM analysis. The mean nanoparticle hydrodynamic diameter was also determined in the presence of serum and it was found to be increased by 5 nm (Supplementary Data, Fig. S2), potentially due to the formation of a soft protein corona [16]. Having demonstrated that cathepsin B efficiently induces digestion of the PGATyr copolymer, it was expected that the presence of the proteolytic enzyme would also induce HPNP digestion resulting in the formation of smaller particles/complexes and the release of free hematoporphyrin. In order to investigate the effect of cathepsin B on the HPNPs, the formulation was incubated with cathepsin B, at pH 7.4 and pH 6.4, at 37 °C, for 24 h. Digested samples were analyzed using DLS and compared to the undigested formulation (Fig. 2c). The mean diameter of the nanoparticles digested with cathepsin B at pH 6.4 was 31 nm, with diameters ranging between 23 and 55 nm, while the corresponding mean diameter of the nanoparticles digested at pH 7.4 was 23 nm, with diameters ranging between 18 and 31 nm and 2–3 nm. The data in Fig. 2c demonstrate that digestion of nanoparticles with cathepsin B for 24 h resulted in 44% and 58–95% decrease of nanoparticle size, at pH 6.4 and pH 7.4, respectively. Surprisingly, the level of digestion at physiological pH was higher than that at pH 6.4 (*p* > 0.05), despite the fact that the *co*-polymer is digested more efficiently at pH 6.4, as shown in Fig. 2a. Importantly, for digesting the nanoparticulate assembly, the endopeptidase activity of the enzyme is sterically more essential than the exopeptidase activity for facilitating effective and rapid digestion. Interestingly, although the optimum pH for the exopeptidase activity of cathepsin B is 5, the endopeptidase activity increases with increasing pH, due to the disruption of the ionic bonds that stabilize binding of the occluding loop to the enzyme body, and the pH optimum is found to be at neutral values [17]. This occurrence can rationalize the more efficient digestion of nanoparticles at pH 7.4 than at pH 6.4. Another explanation is related to the stronger intramolecular hydrogen bonding between the more protonated carboxyl side chains and the polymer backbone at pH 6.4, compared to pH 7.4 [18]. These stronger intramolecular hydrogen bond interactions at pH 6.4 can prevent the dissociation /release of polymer fragments from the main nanoparticulate assembly. On the contrary, the more negatively ionized form of the digestion-produced polymer fragments and nanoparticulate assemblies at pH 7.4 result in stronger electrostatic repulsion between them [18]. These phenomena can also explain the appearance of a peak at 3 nm diameter for pH 7.4, which indicates the detection of small distinctive fragments or polymer-hematoporphyrin aggregates.

It is worth noting that higher concentrations of cathepsin B in the afore-mentioned systems could have potentially resulted in improved levels of digestion and the formation of smaller particles. However, when higher cathepsin B concentrations were used in these experiments, a precipitate of aggregates was formed upon extensive digestion and the subsequent production of highly hydrophobic fragments that distorted DLS measurements. Due to the formation of hydrophobic fragments upon digestion with the enzyme, the systems in the following experiments were set up in the presence of FBS, which prevented aggregation and precipitation of the fragments. The presence of serum is also a more representative condition of biological systems, as distinct from serum-free experimental settings.

Overall, the results demonstrate the efficient cathepsin B-induced digestion of nanoparticles into smaller particles (Fig. 1c), which potentially translates to an improved distribution of the sensitizer in dense tumour structures. This is particularly important considering the impaired nanoparticle diffusion throughout dense tumour masses, which mainly results from the high-interstitial fluid pressure and the dense network of collagen fibers [19]. These results suggest that, upon tumour accumulation through the EPR effect, the nanoparticles can be digested into smaller particles by the increased pericellular cathepsin B secreted by malignant cancers. This may subsequently enhance particle diffusion throughout the dense tumour mass upon extravasation, preventing excess sequestration and entrapment in the perivascular space. Cathepsin B has a pH optimum of 4.5 to 5.5, and it is expected that complete nanoparticle digestion and subsequent hematoporphyrin release occurs within the lysosomes. This occurrence can promote the incorporation of free hematoporphyrin or amphiphilic hematoporphyrin complexes in the lysosomal membrane, the sensitization of the latter and the subsequent lysosome collapse upon ultrasound irradiation, due to sonochemical effects (Fig. 1d) [20]. It has previously been shown that lysosomal collapse can significantly decrease cytoplasmic pH, which can lead to apoptosis [21]. Hence, it is hypothesized that this is one of the potential mechanisms of the treatment modality described in this study and the effect is supported by the responsiveness of the nanoparticulate formulation to cathepsin B.

The surface potential of the nanoparticles was −24.76 ± 1.76 mV at pH 7.4 and − 22.54 ± 2.01 at pH 6.4 (Fig. 2d). The lower absolute zeta potential of the undigested particles at pH 6.4, compared to physiological pH, is explained by the higher protonation level of the glutamate carboxylic acid side chains and the carboxylic acid residues of hematoporphyrin. The data in Fig. 2d indicate that digestion with cathepsin B for 24 h decreased the surface potential to −13.80 ± 1.63 and to −18.36 ± 1.41 at pH 7.4 (*p* < 0.01) and pH 6.4 (*p* > 0.05), respectively. Importantly, the reduction of negative charge upon digestion can lessen charge repulsion between the nanoparticles and cellular membranes for improving cellular uptake.

**Fig. 3:**
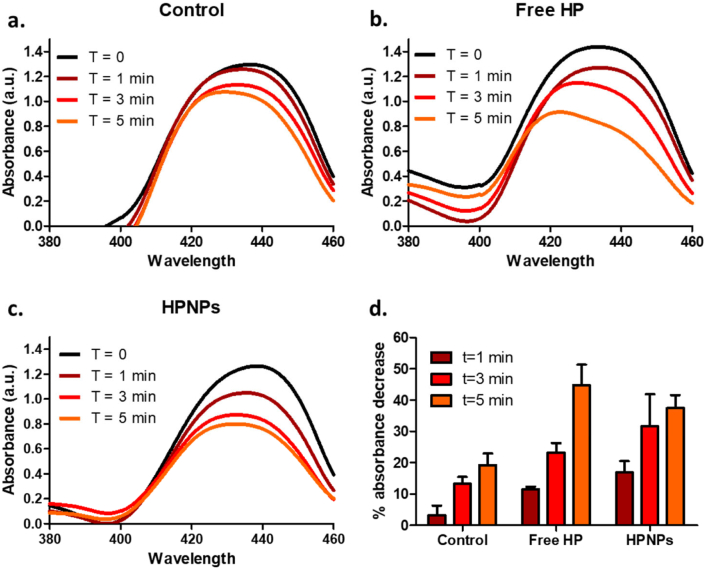
Spectra indicating the time-dependent decrease of DPBF absorbance induced by ROS, in ultrasound-exposed: a.sensitizer-free solvent mixture, b. free hematoporphyrin solution and c. HPNP suspension. Curves are formed using the mean values, with *n* = 3. c. Percentage of absorbance decrease at 436 nm compared to the ultrasound-untreated corresponding samples (n = 3).

The typical production of cytotoxic ROS (except for hydrogen peroxide) during sonodynamic activation using the nanoparticulate formulation was demonstrated using DPBF degradation induced by ROS and confirmed by the consequent decrease in absorbance overtime, within the course of 5 min. For comparative purposes, the production of ROS was also evaluated in the presence of free hematoporphyrin and in the absence of any sensitizing agent, *i.e.* only upon ultrasound irradiation. The data in Fig. 3 demonstrate that, upon ultrasound exposure, the nanoformulation has comparable performance with the free hematoporphyrin (*p* > 0.05), in terms of ROS production, and this is indicative of an efficient SDT-induced antitumour effect.

### 3.3 “Silent” dose-response and the effect of pH

**Fig. 4:**
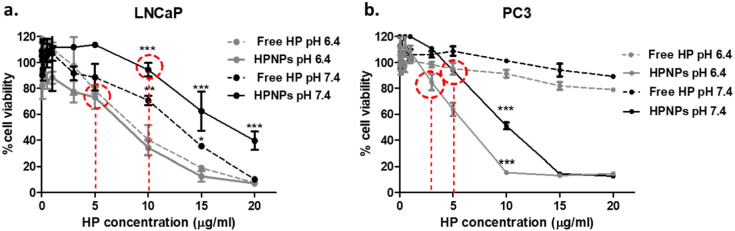
“Silent” cytotoxicity of free hematoporphyrin (Free HP) and HP-containing PGATyr nanoparticles (HPNPs), at pH 7.4 and pH 6.4, for LNCaP (a) and PC3 (b) cells. The concentrations used in following experiments for pH 6.4 and pH 7.4, for each cell line, are indicated in red. (*p < 0.05 and ***p < 0.001, n = 3). The *p* values show the significance of difference between samples incubated at pH 6.4 and those at pH 7.4. (For interpretation of the references to colour in this figure legend, the reader is referred to the web version of this article.)

In order to identify the maximum nanoparticle concentration with low cytotoxicity for subsequent cellular uptake studies and to clearly demonstrate ultrasound-induced cytotoxicity, *i.e.* a sonodynamic effect, in experiments to follow, it was essential to determine the toxicity profile of the nanoparticles and compare it with that of free hematoporphyrin, at physiological and acidic conditions. This would allow us, in each case, to use HPNP and free HP concentrations with minimal toxic effect in the absence of ultrasound (“silent” effect). LNCaP and PC3 cells were treated with increasing concentrations of the free sensitizer or the nanoparticulate formulation, ranging from 0.01–20 μg/mL based on HP concentration. All systems were undertaken in the presence of FBS, therefore aggregation and subsequent precipitation of free HP was avoided. Results in Fig. 4 demonstrate that, for LNCaP cells, the free and nanoparticulate HP elicited “silent” cytotoxicity at concentrations higher than 10 μg/mL at pH 7.4. The corresponding toxicities at pH 6.4 were significantly increased, with the highest comparable concentration of relatively low cytotoxicity identified at 5 μg/mL. The “silent” toxicity profile for PC3 cells was different. The free sensitizer did not have any cytotoxic effect at concentrations up to 20 μg/mL, while the nanoparticulate sensitizer showed significant toxicity at concentrations higher than 3 μg/mL and 5 μg/mL, for pH 6.4 and pH 7.4, respectively. Overall, these results demonstrate that the nanoparticulate formulation had increased cytotoxic effect at acidic pH for both cell lines. This is particularly important considering that improved cytotoxicity at the tumour acidic conditions can confine and potentiate the ablation effect within the tumour mass, reducing the effects on healthy surrounding tissues.

### 3.4 The effect of pH and cathepsin B on cellular uptake

**Fig. 5:**
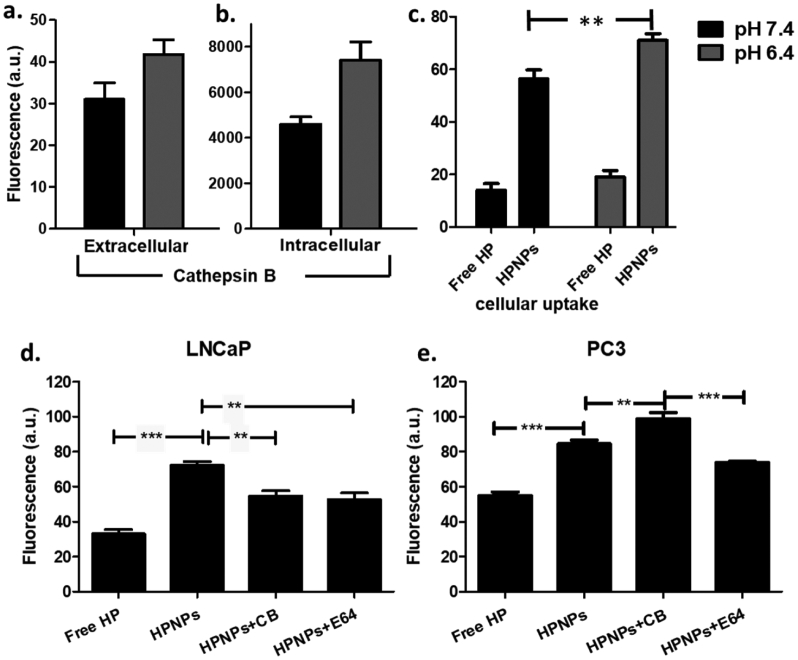
Levels of extracellular (a) and intracellular (b) cathepsin B after 24 h incubation of LNCaP cells at hypoxic conditions, at pH 7.4 and pH 6.4. Cellular uptake (c) after incubation of LNCaP cells with nanoparticles (HPNPs) for 24 h, at the corresponding conditions. Cellular uptake after incubation of LNCaP (d) and PC3 (e) cells with free hematoporphyrin (Free HP), nanoparticles (HPNPs), nanoparticles with cathepsin B (HPNPS+CB) and nanoparticles with chathepsin B inhibitor E64 (HPNPs+E64) for 24 h, at acidic and hypoxic conditions. **p < 0.001 and ***p < 0.0001 (*n* = 4).

The nanoparticulate formulation developed in this study is designed to respond to features of the tumour microenvironment, such as the acidic tumour interstitium and cathepsin B. Cathepsin B is among the proteolytic enzymes that are abundant in the microenvironment of malignant tumours and its expression and secretion by cancer cells are extensively regulated on the basis of the interstitial pH. Examining the intracellular and secreted levels of this enzyme for LNCaP cells was an essential step based on the aim to exploit cathepsin B for improving the performance of our therapeutic platform in SDT. Considering that cathepsin B secretion in tumours is triggered by hypoxia [22], the incubations were carried out under hypoxic conditions, at 2 mmHg O_2_. The results in Fig. 5a and b demonstrate that, for LNCaP cells, at pH 6.4, the levels of extracellular and intracellular cathepsin B were elevated by 35% and 61%, respectively, at acidic pH, when compared to pH 7.4. The cellular uptake of the nanoparticles, at a final concentration of 5 μg/mL with respect to HP concentration, for both pH conditions was determined. HPNP uptake by LNCaP cells was increased by 75% for pH 6.4 when compared with pH 7.4 (Fig. 5c) and this corresponds with the increase in cathepsin B at this pH (Fig. 5b). Although, this is not robust evidence of direct correlation of cathepsin B with nanoparticle cellular uptake, it is suggestive that the levels of the proteolytic enzyme have an effect on cellular uptake of the PGATyr-based nanoparticles. This result may also be attributed to the expected increased protonation of the glutamate residue side chains at acidic conditions that would lead to reduction of the absolute zeta potential, facilitating improved cell internalization.

Since precedence in the literature suggested improved cellular uptake of PGA-based nanoparticles that were digested with cathepsin B [10], the role of cathepsin B, in this context, was further investigated. The uptake of HPNPs by LNCaP cells was examined at pH 6.4 in the presence of extra cathepsin B added in the cell incubation systems and in the presence of E-64, a cathepsin B inhibitor. The corresponding systems were also set up to examine the effect of supplemental cathepsin B and E-64 on HPNP uptake by PC3 cells, at pH 6.4. While the experiment was carried out using a concentration of 5 μg/mL for the free HP and HPNP in the LNCaP experiment, for the PC3 experiment, the free HP or HPNP were used at a final concentration of 3 μg/mL. Data in Fig. 5d demonstrate that the presence of added cathepsin B and E-64 in the incubation systems resulted in 24% and 26% reduction, respectively, in HPNP cellular uptake for the LNCaP cells. Although the presence of cathepsin B had negative impact on cellular uptake for LNCaP cells, in the case of PC3 cells, the supplemental cathepsin B significantly (*p* < 0.01) increased nanoparticle uptake by 17%, while E-64 decreased the uptake by 13% (Fig. 5e). In overall terms these results support the suggestion that cathepsin B is playing some role in enhancing the uptake of the payload by the cells. Importantly, the addition of the cathepsin B inhibitor E-46 did partially inhibit enhanced uptake and we suggest that cathepsin B is certainly involved in the mechanism by which the HPNP uptake is enhanced. In addition, results in Fig. 5 demonstrate that for both cell lines, the nanoparticulate platform significantly increased cellular uptake of hematoporphyrin, since the uptake of the free agent was 54% and 35% lower for LNCaP and PC3 cells, respectively, when compared to the uptake of the nanoparticulate system.

In order to properly interpret the results in Fig. 5c, d and e, it is important to take into consideration that LNCaP cells express prostate specific membrane antigen (PSMA) whereas PC3 cells do not. Similarly to cathepsin B, PSMA exhibits glutamate carboxypeptidase activity [23] and, in principle, it leads to degradation of PGA. Based on these results and considering the similar *in vitro* cathepsin B expression profiles of LNCaP and PC3 cells [24], it is hypothesized that the supplemental cathepsin B led to increased nanoparticle degradation, which was enough to have a detectable positive impact on cellular uptake for the PSMA-lacking PC3 cells. For the PSMA-expressing LNCaP cells, at the *in vitro* conditions employed in this study, the presence of additional cathepsin B did not support cellular uptake. This suggests that some other aspect, possibly PSMA-induced degradation, is contributing to the enhanced uptake of HPNP. The reduction of cellular uptake in the presence of cathepsin B could be a result of the enzymatic action of the latter against PSMA. Interestingly, the protease (carboxypeptidase) domain of PSMA contains a functional and critical glutamate residue, which can be potentially cleaved by cathepsin B, and subsequently inhibit the overall nanoparticle size reduction [25]. Efforts are currently underway in order to further explore these interesting observations. Nevertheless, it must be considered that both cathepsin B and PSMA are indigenous molecules and they are variably expressed throughout prostate tumours; hence, the way these molecules interact and the effect of their interaction on nanoparticle cellular uptake cannot be regulated.

### 3.5 *In vitro* sonodynamic effect

**Fig. 6:**
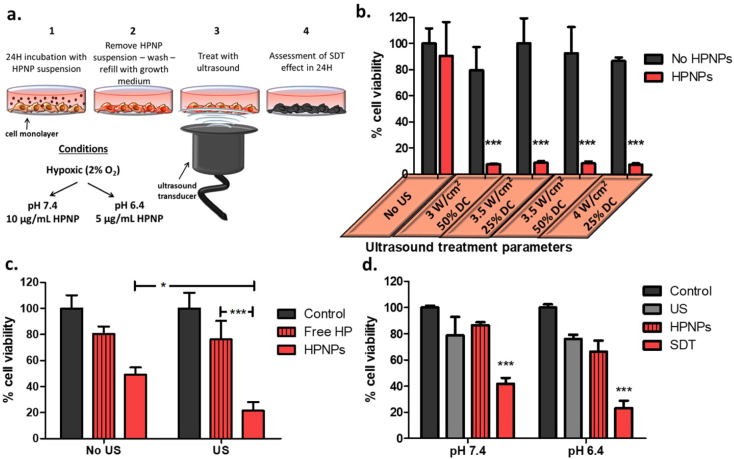
a. *In vitro* treatment protocol. b. % cell viability of LNCaP cells treated with in the absence (No HPNPs) and the presence of nanoparticles (HPNPs), at 10 μg/mL HP concentration, without (No US) and with ultrasound exposure (30 s) at different parameters. c. Cell viability of LNCaP cells treated with no agent (control), free hematoporphyrin (free HP) and nanoparticles (HPNPs), at 5 μg/mL HP concentration, at pH 6.4, without (No US) and with ultrasound (US) exposure at 3 W cm^−2^, 50% DC, for 30 s. d. Cell viability of PC3 cells with no treatment (control), ultrasound only exposure (US), hematoporphyrin nanoparticles nanoparticles at 3 μg/mL (for pH 6.4) and 5 μg/mL (for pH 7.4) HP concentration (HPNPs), and hematoporphyrin nanoparticles in combination with ultrasound (SDT), at pH 6.4 and pH 7.4. Ultrasound exposure was at 3 W cm^−2^, 50% DC, for 30 s. (*p < 0.05, ***p < 0.001, n = 4) For b. and d.:The p values show the significance of difference between samples incubated in the presence and the absence of nanoparticles, under ultrasound exposure.

Having determined that the appropriate nanoparticle concentrations for examining the sonodynamic effect were 10 μg/mL and 5 μg/mL, at pH 7.4 and pH 6.4, respectively, LNCaP cells were treated with the HPNPs and the treatment was combined with ultrasound exposure 24 h later (Fig. 6a). Fig. 6b depicts the effect of HPNPs, at pH 7.4, without and with ultrasound treatment at varying ultrasound parameters, yielding total provided energy between 26 and 60 J/cm^2^. The results also demonstrate the effect of ultrasound exposure on this cell line in the absence of the formulation. The results demonstrate that sonodynamic treatment, *i.e.* ultrasound treatment in combination with the formulation, reduced cell viability by 91–93%, at the ultrasound conditions employed, while exposure to ultrasound alone and the nanoparticles in the absence of ultrasound had no effect on cell viability. This dramatic sonodynamic effect, with no individual ultrasound-induced or nanoparticle-induced “silent” toxicity effects, demonstrates the potential of this technology to achieve efficient and site-specific tumour treatment. In a recent study, Huang and co-workers developed a relatively complex hematoporphyrin-containing nanoparticulate theranostic platform and demonstrated the sonodynamic potential of that system using human breast cancer and lung cancer cell lines [26]. In that study, the treatment, in its non-targeted form, elicited up to approximately 65% *in vitro* toxicity, when irradiated with ultrasound; however, the “silent” effect of the non-targeted form of the nanoparticulate hematoporphyrin was not indicated in the study results, hence it is not possible to appreciate the potential of that system in sonodynamic therapy. The results in Fig. 6b demonstrate a clear ultrasound-induced HPNP toxicity, *i.e.* a clear sonodynamic effect, with no toxicity at the same concentration in the absence of ultrasound.

The sonodynamic performance of the HPNPs against LNCaP cells was also investigated at pH 6.4. Results in Fig. 6c demonstrate that ultrasound irradiation, at 3 W/cm^2^ and 50% DC for 30 s, potentiated the cytotoxicity of HPNPs by 29%. The free hematoporphyrin, at the corresponding concentration, resulted in 20% toxicity, which was not significantly elevated with the application of ultrasound. This finding indicated that, unlike the nanoparticulate hematoporphyrin, the free sensitizer did not elicit a sonodynamic effect under these conditions and this outcome can be potentially attributed to the reduced cellular uptake and the potentially altered subcellular localization, which may affect sonodynamic-induced cytotoxicity. Essentially, this result clearly demonstrates the value of the proposed nanoparticulate platform for enhancing the sonodynamic effect of common hydrophobic sensitizers, such as hematopophyrin. Moreover, for *in vivo* and clinical applications, hematoporphyrin, among other sensitizers, is not recommended for systemic administration as a free agent, due to its hydrophobicity and the subsequent poor biodistribution.

The response of PC3 to the sonodynamic treatment was similarly positive (Fig. 6d), even at the lower HPNP concentrations employed (5 μg/mL for pH 7.4 and 3 μg/mL for pH 6.4), when compared with the LNCaP systems. Results in Fig. 6d demonstrate that exposure to ultrasound augmented HPNP cytotoxicity by 53% and 65%, at pH 7.4 and pH 6.4, respectively. Based on previous reports and the results presented in the current study, different cell lines have shown varying responses to sensitizing agents, the varying formulations of the latter, and the actual sonodynamic treatment [3,27].

### 3.6 *In vivo* SDT treatment

**Fig. 7:**
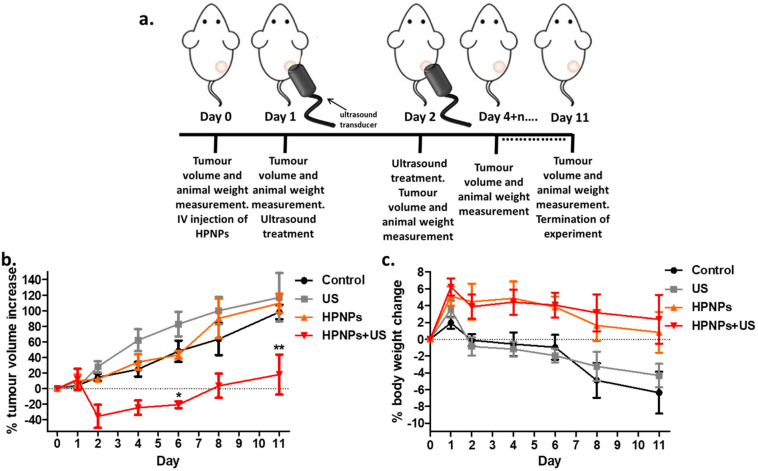
a. *In vivo* treatment protocol. b. Plot of % change of tumour volume treated with (i) no treatment (Control) (ii) ultrasound only (US) (iii) PGA-based nanoparticles carrying hematoporphyrin (HPNPs) and (iv) PGA-based nanoparticles carrying hematoporphyrin with ultrasound, *i.e.* sonodynamic therapy (HPNPs+US). Animals were administered with the formulation on Day 0 and tumours were exposed to ultrasound (3 min) on Days 1 and 2. c. The corresponding animal body weight increase. Error bars represent ± the SD, where *n* = 4.

Having established the sonodynamic performance of the HPNP platform against LNCaP cells *in vitro*, subsequent *in vivo* experiments in SCID mice bearing LNCaP subcutaneous tumours were performed (Fig. 7a). The HPNP formulation was administered intravenously to the tumour-bearing animals at a dose of 6 mg/kg. Approximately 24 h after IV administration of the nanoparticles, tumours were treated with ultrasound. In this study, a higher ultrasound exposure dose was applied compared to the *in vitro* systems, based on previous studies and because of the anticipated tissue attenuation. The data obtained are shown in Fig. 7b and they strongly demonstrate that, while administration of either the HPNPs or ultrasound alone had no impact on tumour growth, treatment using the HPNP formulation in combination with ultrasound, resulted in an average of 36% reduction in tumour volume, within 24 h post- SDT treatment. At that point in time, tumour volume increase for the SDT group ranged from −64% to 3%. Importantly, the SDT-treated tumours reached their initial pre-treatment size on day 8 (where the growth curve crosses the base line), when in control systems, the tumour size had grown beyond the 120% of the initial size. In addition, body weight of the animals administered with the HPNPs formulation was stable, while animals in the other groups (untreated and ultrasound-only treatment) had lost approx. 7% of their body weight by day 11, when the experiment was terminated (Fig. 7c). Interestingly, the animals that were treated with the HPNPs only, without tumour exposure to ultrasound, showed improved body weight increase profile, compared to the other control systems, despite the lack of tumour growth delay. This is a development potentially associated with reduced level of metastatic progression and it requires further investigation. In this study, the administered formulation dose, based on hematoporphyrin concentration, was at similar level and not any higher than the doses used in previous studies [6]. Although, the formulation improved the body weight profile of the animals post-treatment and no adverse effects were observed, future dose escalation studies, accompanied by toxicological analyses, would be necessary in order to determine the dose that can be used to optimize SDT using this formulation.

In a series of recent reports in the literature, SDT has been evaluated for the treatment of a number of different human cancers in preclinical studies, including breast, pancreatic, melanoma, and colon cancer, while no relevant preclinical study has been published for evaluating the effect of this therapeutic approach in the treatment of prostate tumours. Among those studies, sensitizing agents, formulated in varying micro- or nano-particulate systems, were employed. The formulations have included microbubbles (2–3 μm in diameter) with individual molecules of a sensitizer stably attached on their surface [[28], [29], [30], [31]], sensitizer‑gold nanoparticle conjugates [32], and polymeric nanoparticles containing the sensitizing agent [6]. In some of those studies, the sonodynamic treatment was combined with a chemotherapeutic agent, in order to enhance the toxicity of the latter in a site-specific manner and provide an improved therapeutic result based on SDT [33,34]. Regardless of the type of cancer, the sensitizer or the formulation design, the ever-increasing published preclinical studies have clearly demonstrated that SDT affords significant tumour growth delay, with no associated treatment side effects. In order to highlight the improved performance of the formulation described here, it is worth noting that, in any of the previously-published studies, the extent of tumour size reduction did not exceed 20% using nanoparticle-based systems. The results presented here reveal a considerable response of LNCaP prostate tumours to SDT using one single dose of this easily prepared and biodegradable hematoporphyrin-carrying formulation. The exceptional SDT efficacy using this system was not only demonstrated in terms of tumour volume reduction rate, but also in terms of significant tumour growth delay (Fig. 7b). The second ultrasound exposure 48 h after administrating the formulation, did not contribute to any further tumour volume reduction and this indicated the potential elimination of the sensitizing agent from the tumour mass at that length of time. In future studies, the profile of intratumoural accumulation and retention of the formulation need to be characterized with biodistribution studies in order to efficiently plan the optimal time(s) for ultrasound exposure following IV injection. In addition, the tumour growth profile can be used as an indicator to potentially fractionate SDT treatments. In order to properly plan the frequency of repeated treatments, one would need to consider that STD results in the damage and disruption of tumour microvasculature, based on previous findings [35], and that damaged tumour vasculature would lead to poor intratumoural uptake of the formulation after systemic administration. In preclinical studies, the tumour growth profile, post treatment, is a good indicator of vasculature recovery, mainly based on the sustained tumour growth. Although the SDT efficacy, in most studies published thus far, has been examined for one single treatment (single dose of sensitizer formulation and single ultrasound exposure), more recent studies have exploited the affordability of a repeated SDT treatment approach with encouraging results [36]. In the case of prostate cancer and based on the acoustic accessibility of the latter using either extracorporeal or transrectal ultrasound transducer, repeated SDT treatments involve a scenario that could be readily applied in a clinical setting and has the potential to afford complete tumour eradication, in a non-invasive manner and with minimal side effects. Essentially, it is suggested that SDT has significant potential to form the first line treatment for prostate cancer patients, particularly those that do not meet the inclusion criteria for common locoregional ablation approaches, such as high-intensity focused ultrasound (HIFU), cryoablation or irreversible electroporation (IRE). Unlike HIFU, the low-intensity ultrasound employed in SDT allows patients with calcified prostate tissue to be eligible for this treatment. Unlike cryoablation and IRE, SDT does not require the insertion of electrodes or probes inside or in close proximity to the diseased site, allowing treatment with minimum tissue disturbance. For the afore-mentioned reasons, as well as based on the findings of the current study, prostate cancer is among the candidate recalcitrant malignancies, the treatment of which can significantly benefit from SDT.

## 4 Conclusions

In this work, a nanoparticle formulation was developed based on the self-assembly of PGATyr *co*-polymer with HP. The response of the nanoparticles to pH and cathepsin B was investigated and the corresponding performance in SDT treatment was evaluated. The results demonstrated that digestion of the formulation with cathepsin B resulted in the reduction of nanoparticle size and overall negative charge, potentially leading to more efficient diffusion of the nanoparticles throughout impermeable tumour tissues following extravasation. The formulation showed varied “silent” toxicity profiles in the two human prostate cancer cell lines, LNCaP and PC3, while the acidic pH enhanced the nanoparticle toxicity for both cell lines. Cathepsin B expression and secretion levels appeared to be proportional to cellular uptake. Sonodynamic treatment *in vitro* demonstrated a clear ultrasound–induced cytotoxic effect on HPNP-treated prostate cancer cells with little or no effect elicited by either the stimulus (ultrasound) or the formulation in the absence of the stimulus. The nanoparticulate formulation showed ultrasound-induced ROS production comparable to that of the free sensitizer; however, it significantly improved the sonodynamic activity of HP in cell-based systems, primarily as a result of improved cellular uptake. The *in vivo* study in immunodeficient mice, using the nanoformulation, showed a mean 36% reduction in LNCaP tumour volumes within 24 h post-SDT treatment, with the administration of a single nanoparticle dose. No adverse effects were recorded and body weight was stable for the nanoparticle-treated animals. We anticipate that the nanoformulation system described in this study will open new avenues towards the clinical translation of SDT in the treatment of prostate cancer, as well as other recalcitrant cancers, the prognosis and management of which has yet to be improved with currently applied cancer therapies.

The following are the supplementary data related to this article.Fig. S1 a. Plot of pixel brightness along the SDS-PAGE bands for PGATyr samples digested with cathepsin B at pH 7.4 and pH 6.4, over the course of 3 days. A protein MW ladder was used as a reference. The increase in pixel brightness indicates digestion. b. Relative decrease of the mean PGATyr MW. Error bars represent ± the SD (*p < 0.05, ***p < 0.001), where *n* = 3. Fig. S1

Fig. S2 The mean diameter of the main nanoparticulate population in the presence of FBS, at different pH values. Error bars represent ± the SD (*p < 0.05, ***p < 0.001), where n = 3. Fig. S2

Supplementary material Image 1
