# Supplementary figures and images for: Investigating the performance of a novel pH and cathepsin B sensitive, stimulus-responsive nanoparticle for optimised sonodynamic therapy in prostate cancer
Source: J Control Release. 2021 Jan 10;329:76–86. doi: 10.1016/j.jconrel.2020.11.040 (PMC8551370; doi:10.1016/j.jconrel.2020.11.040)

Figure S1

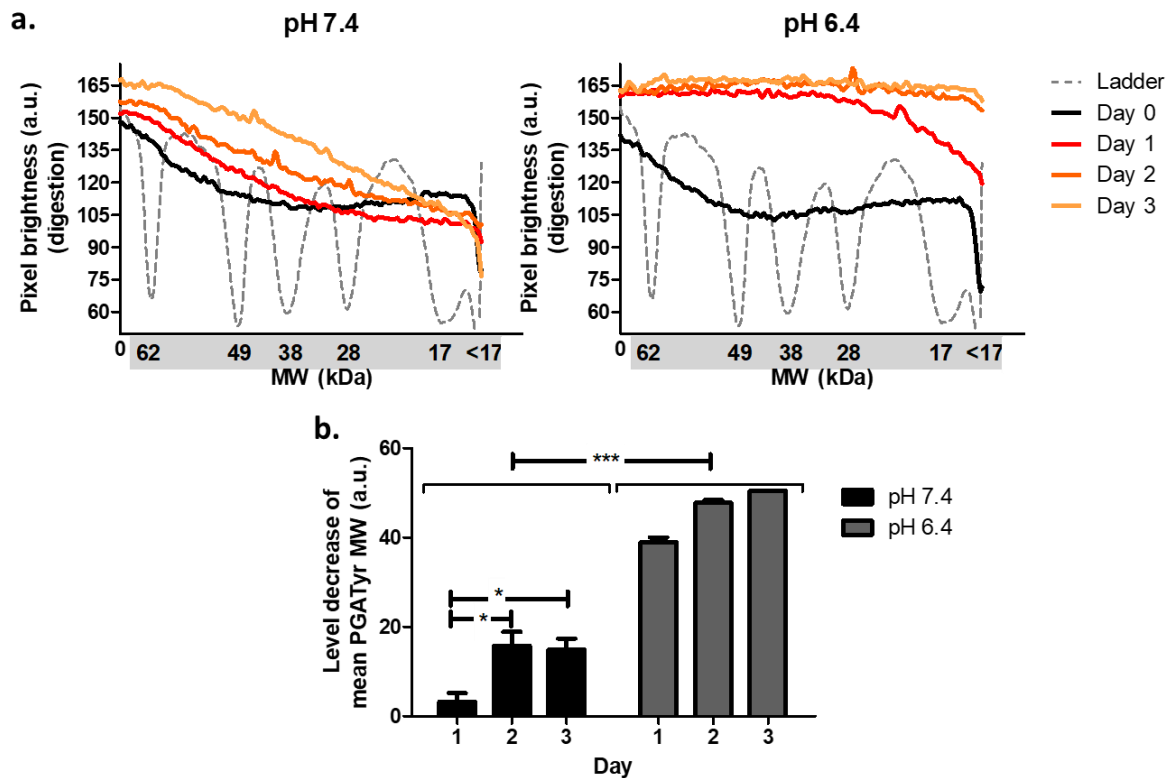

Supplement: The following are the supplementary data related to this article.Fig. S1 — a. Plot of pixel brightness along the SDS-PAGE bands for PGATyr samples digested with cathepsin B at pH 7.4 and pH 6.4, over the course of 3 days. A protein MW ladder was used as a reference. The increase in pixel brightness indicates digestion. b. Relative decrease of the mean PGATyr MW. Error bars represent ± the SD (*p < 0.05, ***p < 0.001), where n = 3. [file mmc1.pdf]

Figure S2

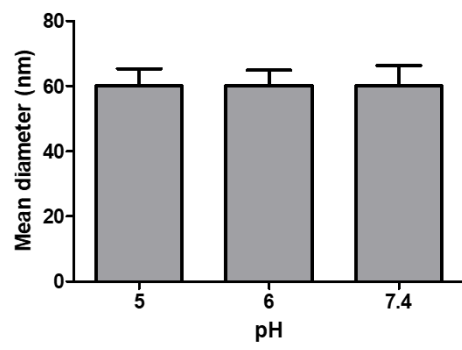

Supplement: Fig. S2 — The mean diameter of the main nanoparticulate population in the presence of FBS, at different pH values. Error bars represent ± the SD (*p < 0.05, ***p < 0.001), where n = 3. [file mmc2.pdf]
