# Supplementary material for: Investigating the performance of a novel pH and cathepsin B sensitive, stimulus-responsive nanoparticle for optimised sonodynamic therapy in prostate cancer
Source: J Control Release. 2021 Jan 10;329:76–86. doi: 10.1016/j.jconrel.2020.11.040 (PMC8551370; doi:10.1016/j.jconrel.2020.11.040)

**Investigating the performance of a novel pH and cathepsin B sensitive, stimulus-responsive nanoparticle for optimised sonodynamic therapy in prostate cancer**

*Marym Mohammad Hadi*^1^, Heather Nesbitt*^2^, Hamzah Masood^1^, Fabiola Sciscione^1^, Shiv Patel^1^, Bala S. Ramesh^1^, Mark Emberton^1^, John F. Callan^2^, Alexander MacRobert^1^, Anthony P. McHale^2^, Nikolitsa Nomikou†^1^*

1. Division of Surgery & Interventional Science, Faculty of Medical Sciences, University College London, UK

2. Biomedical Sciences Research Institute, Ulster University, Coleraine, UK

* Joint first authors

† Corresponding author

**Supplementary Data**

3. Results and Discussion

3.1. Co-polymer digestion with cathepsin-B


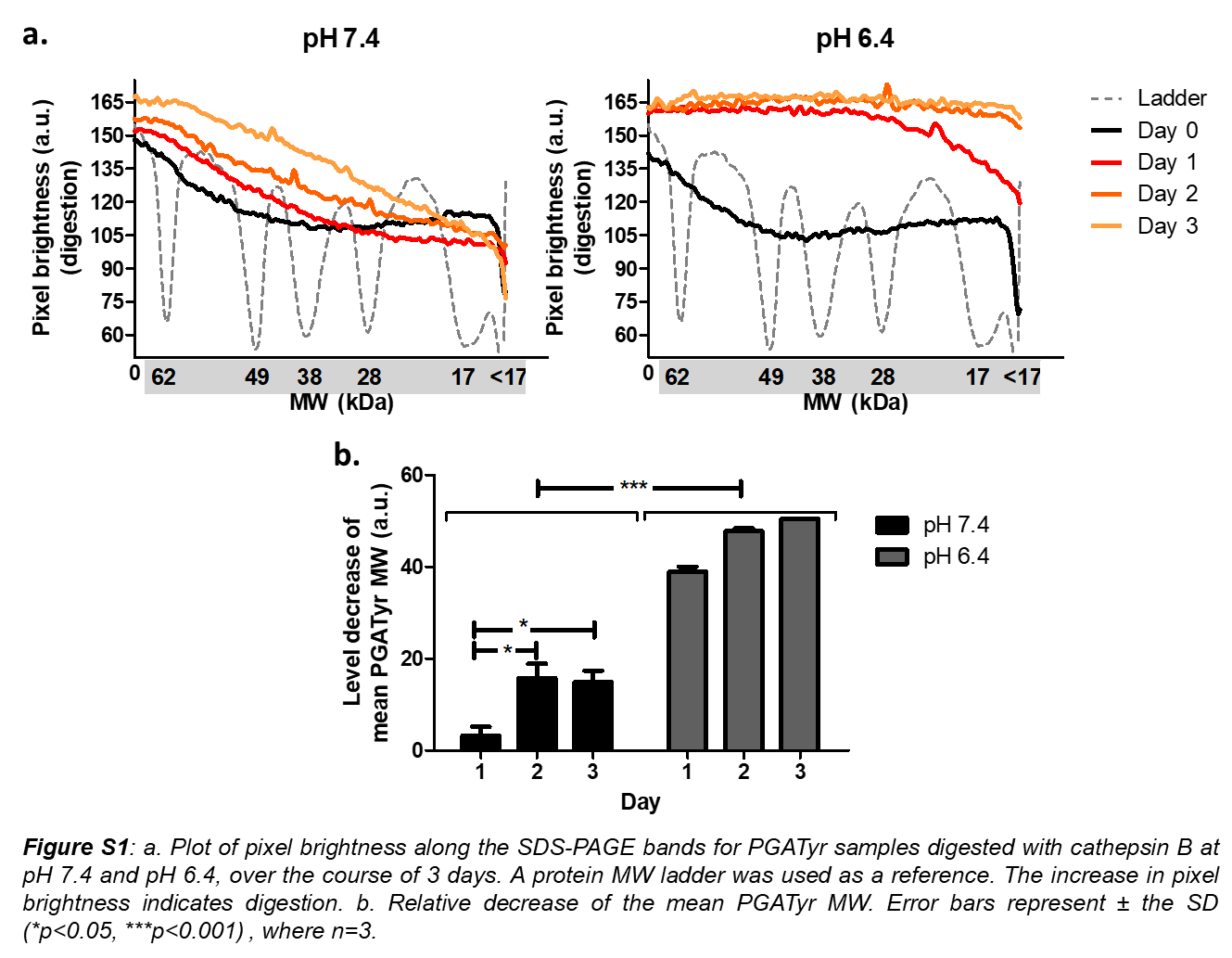


3.2. Nanoparticle characterization and the effect of digestion with cathepsin B


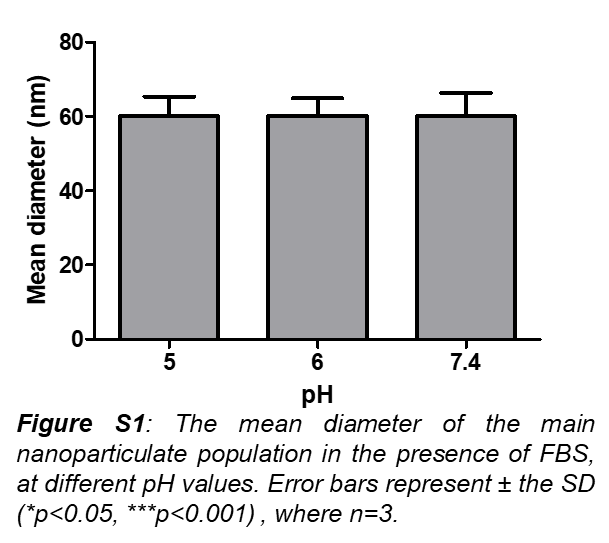

Supplement: Supplementary file 3 — Supplementary material [file mmc3.docx]
